# High-Fructose Diet-Induced Metabolic Disorders Were Counteracted by the Intake of Fruit and Leaves of Sweet Cherry in Wistar Rats

**DOI:** 10.3390/nu11112638

**Published:** 2019-11-03

**Authors:** Kinga Dziadek, Aneta Kopeć, Ewa Piątkowska, Teresa Leszczyńska

**Affiliations:** Department of Human Nutrition and Dietetics, Faculty of Food Technology, University of Agriculture in Krakow, 122 Balicka St., 30-149 Krakow, Poland; aneta.kopec@urk.edu.pl (A.K.); ewa.piatkowska@urk.edu.pl (E.P.); teresa.leszczynska@urk.edu.pl (T.L.)

**Keywords:** Wistar rats, sweet cherry leaves, high fructose diet, lipid metabolism genes, antioxidant enzymes, inflammation

## Abstract

Numerous studies have indicated that the use of plants rich in bioactive compounds may reduce the risk of non-communicable diseases. The aim of this study was to investigate how the addition of fruit and leaves to high-fructose diet affects lipid metabolism, including the expression of genes involved in fatty acid synthesis and oxidation in the liver and adipose tissue, as well as oxidative stress and inflammation in Wistar rats. The animals were fed with AIN-93G diet, high fructose (HFr) diet, HFr diet with addition of 5% or 10% freeze-dried fruits, and HFr diet with addition of 1% or 3% freeze-dried leaves. The experiment lasted 12 weeks. The results showed that the intake of fruit and leaves of sweet cherry caused the improvement of the liver function, as well as beneficially affected lipid metabolism, among others, by regulating the expression of genes associated with fatty acid synthesis and β-oxidation. Additionally, they exhibited antioxidant and anti-inflammatory properties. In conclusion, the addition of fruit and leaves reduced the adverse changes arising from the consumption of high fructose diet. Therefore, not only commonly consumed fruits, but also leaves can be potentially used as functional foods. These findings may be helpful in prevention and treatment of the obesity-related metabolic diseases, especially cardiovascular diseases.

## 1. Introduction

Obesity, recognized as a global epidemic, is becoming a growing global problem. More than 1.9 billion adults, over 340 million children and adolescents aged 5–19, as well as around 41 million children under the age of 5, were overweight or obese in 2016 [1]. Moreover, obesity predisposes to related diseases, such as dyslipidemia, hypertension, non-alcoholic fatty liver disease, and type 2 diabetes.

Literature data indicate that in the last few decades the consumption of fructose has increased. In the United States, in 1977–1978, average daily intake of fructose was 37 g (mainly due to the fresh fruit consumption), in 1988–1994 was 54.7 g and this tendency is still growing. Moreover, Tappy and Lê [2] reported that the world average sugar consumption has increased by around 16% over the 20 years: From 56 g per day per capita in 1986, to 65 g per day per capita in 2007. While, the contribution of fructose in total sugar consumption has increased from around 0% in 1970 to 41% in 2007 [2]. The reason is that fructose, which is sweeter and cheaper than sucrose, is widely used in food industry. High-fructose corn syrup is added not only to sweets, but also to beverages, dairy products, bakery products, and ready to eat meals, i.e., products consumed every day [3,4]. Regular intake of fructose is a factor contributing to the development of lipid disorders, oxidative stress, and chronic mild inflammation, which are directly associated with obesity and cardiovascular diseases [5]. Fructose exhibits pro-oxidative and pro-inflammatory properties by inducing the formation of free radicals, and decreasing non-enzymatic or enzymatic protection, as well as by inducing the production of pro-inflammatory cytokines and activating the nuclear factor kappa-light chain-enhancer of activated B cells (NF-κB), respectively [6,7]. The probable reason of lipid changes lies in the differences in metabolic pathway of fructose and glucose in the liver. Fructolysis bypass the rate-controlling step-phosphofructokinase. The unrestricted fructose metabolism provides the increased amount of substrates for, among others, lipogenesis, resulting in the accumulation of lipids in organs [8]. Additionally, high fructose intake stimulates lipogenesis by affecting the expression of the following transcription factors: *Mlxipl* and *Srebf1.* They regulate the expression of genes involved in lipid synthesis in the liver and adipose tissue: Acetyl-CoA carboxylase (*Acaca*), fatty acid synthase (*Fasn*), and stearoyl-CoA desaturase 1 (*Scd1*). In addition, perturbation of lipid metabolism may also result from the effect of fructose on the expression of peroxisome proliferator-activated receptor α (*PPARα*)-transcription factor responsible for regulating the expression of carnitine palmitoyltransferase 1a (*Cpt1a*) [8].

Numerous studies have shown that dietary interventions, including the consumption of functional foods containing bioactive compounds, have a positive impact on human health. It was reported that antioxidant, anti-inflammatory, and antihyperlipidemic properties of the polyphenols contained in fruit and vegetables can reduce the negative changes caused by high fructose diet [9,10]. Fruits of sweet cherry are seasonal fruits, which are consumed mainly in unprocessed form. The taste, color of the skin, and the content of numerous nutrients and phytochemicals make them attractive to consumers [11]. Literature data have indicated beneficial effect of these fruits on disorders related to non-communicable diseases, among other on lipid metabolism and inflammation [12,13,14]. Our previous studies revealed that not only fruit but also by-product-leaves, unused until now, are a rich source of bioactive compounds and exhibit a high antioxidant activity [15,16]. In available literature, there is lack of in vivo studies concerning potential benefits of sweet cherry leaves as well as studies comparing the fruit and leaves in animals fed with high fructose diet.

Therefore, we hypothesized that the bioactive compounds from fruit and leaves of sweet cherry reduce the adverse changes arising from the consumption of high fructose diet. The aim of this study was to investigate how the addition of these plants to high fructose diet affects lipid metabolism, including the expression of genes involved in fatty acid synthesis and oxidation in the liver and adipose tissue, as well as oxidative stress and inflammation in Wistar rats.

## 2. Materials and Methods

### 2.1. Animals

Growing, male, 6–7-week-old Wistar rats (*n* = 48), initially weighing 131.5 ± 9.6 g, were acquired from Animal Husbandry in Brwinów, Warsaw, Poland. Animals were maintained in plastic cages, at temperature of 22 ± 2 °C, under 12 h/12 h light/dark cycle with free access to food and water. Body weight gain was recorded weekly through the whole experimental period. All experimental procedures were performed in conformity with the Polish Ethical Standards and approved by the First Local Ethical Committee in Krakow Poland (res. no 166/2015; date of approval: 24 June 2015).

### 2.2. Experimental Diets

After one week for acclimation, the rats were randomly divided into six groups (*n* = 8). The experimental diets were prepared based on AIN-93G diet [17]. In high fructose (HFr) diet, the content of starch and sucrose was replaced by fructose, as the carbohydrate source (Table 1). The HFr diet was modified with the addition of fruit and leaves of sweet cherry (*Prunus avium* L.).

The fruit of cultivar Kordia and the leaves of cultivar Regina, which were harvested together at commercial maturity of fruit, were used in this study. They were collected from the Experimental Station of Pomology and Apiculture Department, University of Agriculture in Krakow, in Garlica Murowana, Poland, in 2016. Our previous study revealed that these cultivars had the highest content of total polyphenolic compounds, and they were characterized by the highest antioxidant activity compared to other cultivars. Additionally, in order to balance the composition of experimental diets, the basic chemical composition of fruit and leaves was measured [15]. The fruit and leaves were washed and dried. Additionally, the fruit was cut in half and pitted. Next, they were frozen at −80 °C and freeze-dried in lyophilizer (Christ Alpha 1–4 (Osterode am Harz, Germany)), ground and added to experimental diets in the form of powder.

The rats of the group I received AIN-93G diet-negative control (NC) diet, the group II–HFr diet (positive control diet), the group III–HFr diet with the addition of 5% of fruits (HFr + F5%), group IV–HFr diet with 10% of fruits (HFr + F10%), group V–HFr diet with 1% of leaves (HFr + L1%), as well as group VI–HFr diet with 3% of leaves (HFr + L3%) (Table 1). The level of the addition of sweet cherry fruit and leaves to experimental diets was chosen based on our previous study [18]. The diets of groups III–VI were balanced, taking into account the content of dietary fiber in fruit and leaves of sweet cherry because of its high level [15].

### 2.3. Biological Sample Collection

At the end of the 12-week experimental period, fasted rats were anaesthetized by inhalation of 4% izofluran (Baxter Polska Sp. z o.o., Warsaw, Poland). The blood, taken from the heart by cardiac puncture, was collected into the test tubes with heparin (to obtain whole blood), as well as into the centrifuge tubes (to obtain the serum). The serum was obtained by centrifugation at 1500 rpm for 15 min and stored at −80 °C for further analysis. The liver, heart, kidneys, and visceral (perirenal) adipose tissue were dissected and washed using a cold solution of 0.9% sodium chloride, dried, and weighed. They also were stored at −80 °C.

### 2.4. The Concentration of Crude Lipid and Fatty Acids Composition in Organs and Adipose Tissue

The content of the crude lipid in the liver, heart, and kidneys was measured by Soxhlet method using Soxtec Avanti’s 2050 Auto Extraction Unit (Tecator Foss, Hillerød, Sweden) as previously reported by Kopeć et al. [19]. The fatty acids composition was measured by gas chromatography–mass spectrometry using GC-17A-QP5050 GC-MS model (Shimadzu, Kioto, Japan) according to Kopeć and Piątkowska [20]. Results were shown as the percentage of all fatty acids.

### 2.5. Biochemical Analysis

The concentration of glucose was determined in the whole blood with a glucometer (GlucoSense pro, Genexo Sp. z o.o., Warsaw, Poland).

In the serum, the activity of alanine aminotransferase (ALT) and aspartate aminotransferase (AST) was measured using ALT (GPT) DST kit (cat. no. A6624-050) and AST (GOT) DST kit (cat. no. A6661-050), recpectively (Alpha Diagnostic, Warsaw, Poland). The content of total cholesterol (TC), HDL-cholesterol (HDL) and triglycerides (TG) was determined using the following kits: Liquick Cor-CHOL60 (cat. no. 2-204), Cormay HDL (cat. no. 2-052), and Liquick Cor-TG60 (cat. no. 2-253), respectively (PZ Cormay S.A., Lublin, Poland). The LDL+VLDL level was calculated by the Friedewald formula [21].

In the serum, total antioxidant status (TAS) was measured using Antioxidant Assay kit (cat. no. 709001, Cayman Chemical Company, Ann Arbor, MI, USA). The activity of superoxide dismutase (SOD) was assessed in the erythrocyte lysate, glutathione peroxidase (GPx) in whole blood, and glutathione reductase (GR) in the serum with RANSOD kit (cat. no. SD 125), RANSEL kit (cat. no. RS 504), and GLUT RED kit (cat. no. GR 2368), respectively (Randox Laboratories Ltd., Crumlin, UK). The activity of catalase (CAT) was determined in the serum using Rat Cat Elisa kit (cat. no. ER0264, Wuhan Fine Biotech Co., Ltd., Wuhan, China).

The activity of heme oxygenase-1 (HO-1) was assessed using Liquick Cor-BIL Total 60 kit (cat. no. 2-245, PZ Cormay S.A., Lublin, Poland). Results were expressed as µmol of bilirubin per L. In the serum, the level of thiobarbituric acid reactive substances (TBARS) was measured as previously reported by Ohkawa et al. [22]. The results were shown as nmol of malondialdehyde (MDA) per mL.

The concentration of C reactive protein (CRP) was determined using Rat CRP Elisa kit (cat. no. ER 0016, Wuhan Fine Biotech Co., Ltd., Wuhan, China). In the serum, the level of interleukin-6 (IL-6), tumor necrosis factor (TNF-α), and interleukin-10 (IL-10) was measured with Interleukin-6 (rat) Elisa kit (cat. no. EIA-4845), TNF-α (rat) Elisa kit (cat. no. EIA-4774), and Interleukin-10 (rat) Elisa kit (cat. no. EIA-4773), respectively (DRG Instruments GmbH, Marburg, Germany).

### 2.6. Gene Expression

Total RNA from frozen liver and adipose tissue was isolated with Total RNA Mini Plus kit as well as Total RNA Mini kit, respectively (cat. no. 031-100 and 036-100, respectively, A&A Biotechnology, Gdynia, Poland), following the manufacturer’s protocols. RNA was reverse transcribed to the complementary DNA using the TranScriba kit (cat. no. 4000-100, A&A Biotechnology, Gdynia, Poland). The quantity of RNA and cDNA was measured with the Thermo Scientific μDrop Plate using spectrophotometer (Multiskan GO; Thermo Fisher Scientific Corporation, Vantaa, Finland). cDNA samples were stored at −20 °C until real-time PCR analysis. The PCR reaction mixture contained cDNA samples, RNase-free water, TaqMan Gene Expression Master Mix (Applied Biosystems, Foster City, California, USA), and primers for following genes: Fatty acid synthase (*Fasn*), acetyl-CoA carboxylase (*Acaca*), stearoyl-CoA desaturase 1 (*Scd1*), MLX-interacting protein-like (*Mlxipl)* (known also as Carbohydrate-responsive element-binding protein (*ChREBP*)), and additionally in the liver on factors: Factor sterol regulatory element binding protein 1 (*Srebf1*), carnitine palmitoyltransferase 1a (*Cpt1a*), and peroxisome proliferator-activated receptor alpha (*Ppar-α*) with fluorescent marked starters. The reaction mixture was incubated for 2 min at 50 °C and for 10 min at 95 °C (initial step), as well as 40 cycles of 95 °C for 15 s (denaturation) and 60 °C for 1 min (annealing/extension) using CFX96 Touch™ Deep Well Real-Time PCR Detection System (Bio-Rad, Hercules, CA, USA). The reference gene was β-actin (*Actb*). The results were determined using the 2^−ΔΔC^T method as previously reported by Livak and Schmittgen [23].

### 2.7. Statistical Analysis

Results were expressed as means ± SD (*n* = 8). Normality was assessed with the Shapiro–Wilk test. Based on obtained results, one-way analysis of variance (ANOVA) was carried out. Duncan post-hoc test was used to determine significant differences between mean values (*p* < 0.05). All statistical analyses were performed using software Statistica v. 13.1 (Tulsa, OK, USA).

## 3. Results

### 3.1. Body Weight Gain and Weight of Organs

Statistically significant differences in body weight gain between groups of rats were not found (Table 2). The weight of the liver of animals fed with HFr + F10% and HFr + L1% diets was significantly lower in comparison with the weight of liver of rats receiving HFr diet. Rodents receiving HFr + F10% diet were characterized by the highest heart weight, however, rats fed with the HFr + F5% diet were characterized by the lowest. The weight of kidneys of rats receiving HFr and HFr + L3% diets was significantly higher compared to weight of kidneys of animals fed with NC diet.

### 3.2. Crude Lipid Content and Concentration of Fatty Acids in Selected Organs and Adipose Tissue

The rats fed with HFr + F10%, HFr + L1%, and HFr + L3% diets were characterized by the significantly lower content of crude lipid in the liver in comparison with rodents receiving HFr diet (Table 2). Importantly, the addition of fruit and leaves to HFr diet allowed for obtaining values similar or even lower to those found in rats fed with NC diet.

The addition of sweet cherry fruit and leaves to HFr diet did not affect the level of total SFA, MUFA, and PUFA in the livers (exception was the level of SFA in the livers of rats fed with HFr+F5% compared to the livers of animals receiving HFr diet) (Table S1). However, intake of HFr+F5%, HFr + F10%, HFr + L1%, and HFr + L3% diets decreased the level of C14:1 and C24:0 in comparison to HFr diet. Additionally, the livers of rats fed with HFr + F10% and HFr + L3% were characterized by the lowest content of C15:0 and C20:3 (*n-6*), respectively, in comparison to other animals. C24:0 was detected only in kidneys of rats fed with NC diet (Table S2). Meanwhile, the kidneys of animals fed with HFr, HFr+F5%, HFr + F10%, HFr + L1%, and HFr + L3% diet contained C20:2 (*n-6*), which were not detected in kidneys of rodents receiving NC diet. HFr diet increased the concentration of total PUFA (especially C18:2 (*n-6*), C20:4 (*n-6*) and C20:5 (*n-3*)) in comparison to NC diet. The addition of sweet cherry fruit and leaves to the HFr diet did not change the ratio of SFA, MUFA, and PUFA in kidneys of rats. It was only found the slight differences in the quantity of individual fatty acids.

Dietary treatment did not affect the level of total SFA, MUFA, and PUFA in heart of rats (Table S3). However, the hearts of animals fed with HFr diet with the addition of fruit had higher content of C18:3 (*n-6*) as well as lower content of C15:0 and C22:6 compared to hearts of rats receiving HFr diet. It was also observed the lower amount of C22:6 in hearts of rodents fed with HFr + L1% and HFr + L3% diets. Additionally, HFr + L1% diet decreased the level of C16:1 and C20:2 (*n-6*), as well as HFr + L3% diet reduced the content of C12:1 and C14:2, in comparison to HFr diet.

HFr diet caused a significant increase in the content of C18:1 (*n*-9), thereby total MUFA, in adipose tissue of rats in comparison to NC diet (Table S4). The addition of fruit and leaves to HFr diet did not affect the fatty acid profile in adipose tissue compared to HFr diet.

### 3.3. Selected Biochemical Parameters

The use of experimental diets did not cause the differences in glucose levels in whole blood of rats (Table 2). The animals fed with HFr and HFr + F10% diets were characterized by the significantly highest activities of ALT and AST in the serum. Whereas, HFr+F5%, HFr + L1%, and HFr+F3% diets resulted in the decrease in the level of these enzymes compared to HFr diet. It should be pointed out that the intake of these diets allowed for decreasing AST activity below the level recorded in NC group. Dietary treatment did not affect the concentration of TC in the serum of animals (Table 2). However, we observed the significant increase in the level of HDL cholesterol in rats fed with HFr + F5%, HFr + L1%, and HFr+F3% diets, as well as decrease in the concentration of LDL+VLDL cholesterol in animals receiving the HFr + F10% diet, in comparison to HFr diet. The rodents fed with HFr + F10% and HFr + L1% diets had lower levels of TG in the serum compared to rats receiving HFr diet.

### 3.4. Biochemical Parameters Related to Oxidative Stress and Inflammation

The addition of fruit and leaves to HFr diet did not change the TAS level in comparison to HFr diet, whereas it affected the activities of antioxidant enzymes (Table 3). The intake of HFr + F5%, HFr + F10%, HFr + L1%, and HFr + L3% diets resulted in significant increase in the level of GPx and decrease in the level of CAT compared to HFr diet. It is worth mention that the decreased CAT activity reached the similar or even lower level than in animals receiving NC diet. Additionally, the animals fed with HFr + L3% had significantly lower activity of SOD, however, the rats receiving HFr + F10% had the higher level of GR, in comparison to rodents receiving HFr diet. The effect of dietary treatment on HO-1 concentration in the serum of animals was not found. The level of TBARS was significantly lower in the serum of rats receiving HFr + F5% diet, compared to animals fed with HFr diet.

The rats fed with HFr + F5%, HFr + F10%, HFr + L1%, and HFr + L3% diets were characterized by the lower concentration of CRP in the serum in comparison with animals receiving HFr diet (Table 3). It should be noted that above-mentioned CRP concentrations were on similar level compared to those found in NC group. The highest level of IL-10 was observed in the serum of rats fed with HFr + F10% and HFr + L3% diets compared to NC and HFr diets.

### 3.5. Gene Expression Analysis

**Figure 1:**
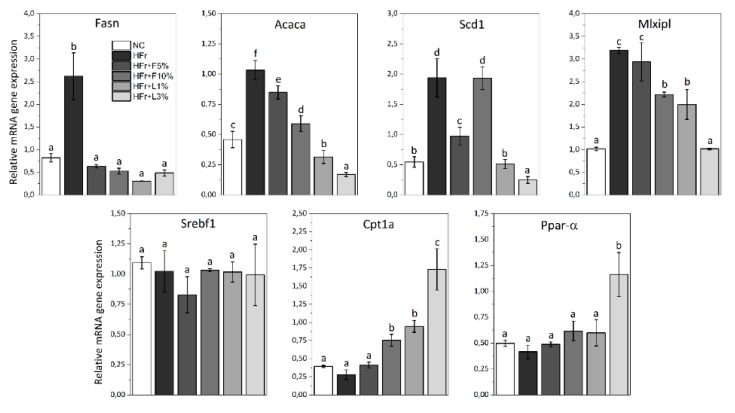
The expression of fatty acid synthesis genes (*Fasn, Acaca, Scd1, Mlxipl* and *Srebf1*) and fatty acid oxidation genes (*Cpt1a* and *Ppar-α*) in liver of rats. NC—negative control; HFr—high fructose; HFr + F5%—HFr diet with addition of 5% of fruits; HFr + F10%—HFr diet with addition of 10% of fruits; HFr + L1%—HFr diet with addition of 1% of leaves; HFr + L3%—HFr diet with addition of 3% of leaves. Data are expressed as mean ± SD. Values with different letters (a–f) are statistically different (*p* < 0.05).

The intake of HFr diet significantly increased the mRNA expression of the following fatty acid synthesis genes in the liver: *Fasn*, *Acaca*, *Scd1,* and *Mlxipl*, in comparison to NC diet (Figure 1). In the liver of rats fed with HFr + F5%, HFr + F10%, HFr + L1%, and HFr + L3% diets, expression of *Fasn*, *Acaca*, *Scd1*, and *Mlxipl* was significantly lower compared to animals receiving the HFr diet (exception was the expression of *Scd1* in rodent fed with HFr + F10% diet, as well as expression of *Mlxipl* in rats receiving HFr + F5% diet). It should be emphasized that these values (especially in groups fed with HFr + L1% and HFr + L3% diets) reached the similar or even lower level than in rats receiving NC diet. Additionally, HFr + L3% diet resulted in the significantly highest decrease in *Acaca*, *Scd1*, and *Mlxipl* expression compared to other groups.

The addition of sweet cherry fruit and leaves increased the expression of fatty acid oxidation genes: *Cpt1a* and *Ppar-α (*Figure 1*)*. *Cpt1a* expression was significantly increased in the liver of rats fed with HFr + F10%, HFr + L1%, and HFr + L3% diets compared to rodents fed with HFr diet. In addition, the intake of HFr + L3% diet resulted in the significantly highest increase in *Cpt1a* and *Ppar-α* expression in the liver in comparison to HFr diet.

**Figure 2:**
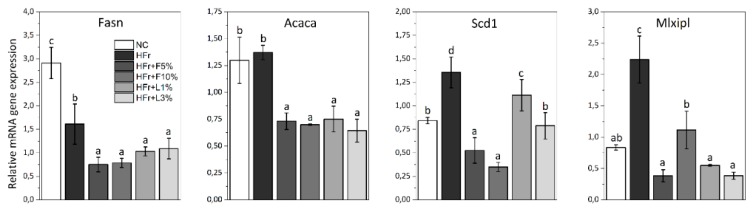
The expression of fatty acid synthesis genes (*Fasn, Acaca, Scd1, Mlxipl*) in adipose tissue of rats. NC—negative control; HFr—high fructose; HFr + F5%—HFr diet with addition of 5% of fruits; HFr + F10%—HFr diet with addition of 10% of fruits; HFr + L1%—HFr diet with addition of 1% of leaves; HFr + L3%—HFr diet with addition of 3% of leaves. Data are expressed as mean ± SD. Values with different letters (a–d) are statistically different (*p* < 0.05).

The consumption of HFr diet significantly increased the expression of *Scd1* and *Mlxipl* in the adipose tissue of rats compared to NC diet (Figure 2). The expression of *Fasn*, *Acaca*, *Scd1*, and *Mlxipl* in adipose tissue of animals fed with HFr diet with addition of fruit and leaves was significantly lower than in rats receiving HFr diet. The highest reduction of *Scd1* expression was observed in rodents fed with HFr + F5% and HFr + F10% diets, while the highest reduction of *Mlxipl* expression was noted in rodents receiving HFr + F5%, HFr + L1%, and HFr + L3% diets. The values reached similar or even lower levels than those observed in the NC group.

## 4. Discussion

In our study, for the first time, the effect of addition of sweet cherry leaves to high fructose diet on the biochemical parameters related to lipid metabolism (including analysis of gene expression), oxidative stress and inflammation in rats was explored. What is more, in available literature there is a limited data on the role of sweet cherry fruit in high-fructose diet-induced metabolic disorders. Therefore, the discussion is an attempt to explain the obtained results.

The use of experimental diets did not affect body weight gain of rats. However, the effect on the other parameters related to obesity and other non-communicable diseases was observed. The HFr diet caused the deterioration of the liver function, which was manifested by an increase in its weight and crude lipid content, as well as increase in the activity of ALT and AST in comparison to rats fed with NC diet. The other authors obtained the similar results [24,25].

It is well known that chronic intake of fructose stimulates de novo lipogenesis, leading to an increase in synthesis and accumulation of lipid in the liver [8]. Additionally, dietary fructose causes changes in serum lipid profile, mainly an increase in triglycerides level and decrease in HDL cholesterol concentration, which has also been observed in our study. Other authors reported the similar results [24,25]. One of the reasons may be the increase in de novo lipogenesis induced by the liver metabolism of fructose. This disturbance can also be associated with the decrease in lipoprotein lipase activity. Intake of fructose does not stimulate insulin secretion, which is known as a factor regulating the activity of lipoprotein lipase in the adipose tissue. Consequently, the lack of changes in insulin concentration during fructose intake does not affect lipoprotein lipase activity, which may lead to hypertriglyceridemia [26]. What is more, fructose-induced metabolic dyslipidemia is usually accompanied by body insulin resistance, which is another key factor contributing to metabolic syndrome development [27]. The literature data indicates that high fructose diet stimulates lipogenesis also by affecting the expression of the genes involved in fatty acid synthesis: *Fasn*, *Acaca*, *Scd1*, and *Mlxipl* [5,28], as well as β-oxidation: *Ppar-α* and *Cpt1a* [8]. The results of our study confirmed these findings.

On the other hand, we observed that the addition of sweet cherry fruit and leaves to HFr diet resulted in an improvement of the liver function and hypolipidemic effect. Rodents receiving HFr + F5%, HFr + L1%, and HFr + L3% diets had significantly lower activity of ALT and AST in the serum. However, the activities of ALT and AST did not change significantly in rats fed with HFr + F10% compared to animals receiving HFr diet. We suppose that this effect is related to the additional content of simple sugars (including fructose) in the diet, provided with fruit of sweet cherry. The lipid content in the liver of animals fed with HFr + F10%, HFr + L1%, and HFr + L3% diets was significantly lower in comparison with the liver of rats receiving HFr diet. The addition of fruit and leaves to the HFr diet also caused improvement of the lipid profile in the serum (Table 2). Der Werf et al. [13] observed the beneficial effect of consumption of sweet cherry fruit in rats fed with high-fat/high-fructose diet, i.e., decrease in the hepatic lipid accumulation, as well as reduction in cholesterol and triglycerides level in the serum. We suppose that the bioactive compounds contained in fruit and leaves of sweet cherry (dietary fiber, carotenoids, polyphenols) [15,16] can be responsible for this effect. The fruits contain mainly a soluble fiber [29]. Literature data indicate that its hypolipidemic effect may be related to an increase in the fecal excretion of cholesterol, triglycerides, and bile acid [30,31]. Additionally, the soluble fiber affects beneficially the activity of hepatic 3-hydroxy-3-methyl-glutaryl-coenzyme-A (HMG-CoA) reductase and lipoprotein lipase, which play an important role in regulating the level of serum cholesterol and triglycerides, as well as the accumulation of lipids in organs [31].

We assume that the beneficial effect on lipid metabolism, which was reported to be more significant in rats fed with diets with the addition of leaves, is mainly due to the presence of polyphenolic compounds which exhibit the pleiotropic effect [32]. Our results showed that leaves were richer source of these compounds than fruit, mainly phenolic acids such as chlorogenic acid and caffeic acid [16]. Therefore, the role of these compounds in regulating lipid metabolism should be discussed with particular emphasis. It is known that chlorogenic acid stimulates phosphorylation of AMP-dependent kinase (AMPK)—an essential step for activation of kinase. AMPK phosphorylation reduces the activity of acetyl-CoA carboxylase, resulting in inhibition of de novo fatty acid synthesis, the decrease in circulation of free fatty acids, as well as the decrease in concentration of cholesterol and triglyceride in the serum [33]. Moreover, chlorogenic acid reduces the activity of glucose-6-phosphatase-last enzyme of the gluconeogenesis, what leads to inhibition of glucose production, as well as, indirectly, to reduction of the synthesis of low density lipoprotein in hepatocytes and to reduction of lipid accumulation in the liver [34]. Additionally, caffeic acid inhibits HMG reductase activity, which results in a reduction of cholesterol levels, including LDL cholesterol [35]. Varghese and Thomas [10], who studied the effect of mulberry leaves extract (also rich in chlorogenic acid) on non-alcoholic fatty liver disease in rats fed with high fructose diet, have observed the similar dependencies. Ibitoye and Ajiboye [36], as well as Yeh et al. [35], reported that the addition of caffeic acid to high fructose and hypercholesterol diet, respectively, affects beneficially lipid profile and liver function.

To explore the molecular mechanisms of the action of sweet cherry fruit and leaves on the lipid metabolism, the expression of selected genes was studied. Our results indicated that the addition fruit and leaves to HFr diet caused significant decrease in expression of *Fasn*, *Acaca*, *Scd1*, and *Mlxipl* (genes of fatty acid synthesis) in the liver and adipose tissue, as well as increase in *Cpt1a* and *Ppar-α* (genes of fatty acid oxidation) in the liver (Figure 1 and Figure 2). Gibert-Ramos et al. [37], who assessed the effect of sweet cherry fruit on lipid metabolism and fat accumulation in Fischer rats treated with 1:1 glucose:fructose solution in water, have also observed that intake of fruit downregulated the expression of *Fasn* and *Acaca* in the adipose tissue. Der Werf et al. [13] have shown that the addition of fruit to high-fat/high-fructose diet caused the reduction in hepatic lipogenic transcription factors: *ChREBP* (*Mlxipl)* and *Srebp*. We suppose, that anthocyanins contained in fruit may be responsible for this activity. Our previous study [15] showed that cyanidin 3-glucoside and cyanidin 3-rutinoside are a main anthocyanins in fruit of cultivar Kordia, which was used in this in vivo study. Chang et al. [38] and Tang et al. [39] assessed the effect of anthocyanins extracts from mulberry, as well as bilberry and black currant, respectively, on lipid metabolism. They reported that anthocyanins extracts, where cyanidin 3-glucoside was a major component, caused the decrease in expression of *Fas*, *Acc,* and *Srebf1*, as well as increase in expression of *Ppar-α* and *Cpt1a*, affecting fatty acid synthesis and β-oxidation in HepG2 cells and in the liver of mice, respectively. This suggests that anthocyanins may have a beneficial effect on lipid metabolism by affecting the expression of the genes.

To the best of our knowledge, there are no studies on the effect of sweet cherry leaves or leaves from other fruit trees on the expression of genes related to lipid metabolism. However, Liao et al. [40], Wan et al. [41], Ilavenil et al. [42], and Naowaboot et al. [43] reported the beneficial effect of individual phenolic acids, i.e., caffeic acid, chlorogenic acid, *p*-coumaric acid, and ferulic acid, respectively, on expression of genes of fatty acid synthesis and oxidation. It may suggest that above-mentioned polyphenolic compounds found also in leaves of cultivar Regina [16] can be responsible for this biological activity.

Additionally, the regulation of lipid metabolism may result from the presence of polyunsaturated fatty acids in cherry, especially in leaves, which are also known from inhibition of expression of the *Srebf1* and its target genes such as *Acc*, *Fas*, and *Scd1* [15,44].

The regulation of genes expression involved in lipid metabolism resulted in improvement of the serum lipid profile and liver function, decrease in lipid accumulation, as well as changes in fatty acid profile in organs. We observed the tendency to decrease in the content of C16:1 and C18:1 in the adipose tissue of rats fed with HFr + F5%, HFr + F10%, HFr + L1%, and HFr + L3% diets compared to HFr diet (Table S4). The explanation for these findings can be associated with the reduction of *Scd1* expression, which converts SFA to MUFA. Other changes in fatty acid profile can suggest that polyphenolic compounds may activate the elongases and desaturases, which are involved in synthesis of particular fatty acids.

The intake of high fructose diet induces oxidative stress [7], which was also observed in our study. It can be caused by the deterioration of the antioxidant defense system, including antioxidant enzymes. Our results showed that the addition of fruit and leaves to HFr diet may counteract this effect (Table 3). The activity of GPx was found to be significantly higher in rats fed with HFr + F5%, HFr + F10%, HFr + L1%, and HFr + L3% diets. The activity of GR tended to be higher in animals receiving these diets (exception was group fed with HFr + F10%, where we observed the significant changes). This is probably related to the antioxidant activity of polyphenolic compounds contained in these plants. Vinitha et al. [45] and Wu et al. [46] also reported higher activity of GPx in mice with streptozotocin-induced neurotoxicity which received ethanolic extract of sweet cherry fruit, as well as in mice fed with high fat diet enriched with purified sweet cherry anthocyanins, respectively, in comparison to control groups. In addition, Šarić et al. [47] showed the similar relationships in mice fed with sour cherry juice. However, in contrast to the above-mentioned studies, we have found a decrease in SOD and CAT activity, especially in the groups of animals receiving diet with the addition of leaves. These observations may be explained by the reduced demand of endogenous enzyme (SOD, CAT) when antioxidants from food are provided. The highest decrease in activity of these enzymes was observed in rats fed with HFr + L3% diet (Table 3), which provided the largest amount of polyphenols. It suggests that the appropriate dose of these compounds may combat free radicals by itself, and not by affecting the first line defense antioxidants. The similar conclusions were made by Estruel-Amades et al. [48]. Our previous studies have shown that fruit and leaves of sweet cherry contained not only polyphenols, but also carotenoids and vitamin C [15,16], which are also known from their antioxidant properties. Our results showed also that the addition of sweet cherry to HFr diet inhibited the formation of TBARS (Table 3). Literature data suggests that polyphenols, among others: Caffeic acid, *p*-coumaric acid, ferulic acid, and anthocyanins, may be responsible for decreasing lipid peroxidation [35,36,49]. All these results indicated that intake of fruit and leaves of sweet cherry beneficially affected the antioxidant status, inhibited the low-density lipoproteins (LDL) oxidation, and thus improved lipid profile.

In addition, imbalance in prooxidant and antioxidant systems is associated with mild chronic inflammation [50]. We observed significantly higher level of CRP and lower TAS in serum of rats fed with HFr diet in comparison to NC diet, confirming these dependencies. Moreover, a reverse relationship was found in animals receiving HFr diet with the addition of fruit and leaves of sweet cherry (Table 3). Anti-inflammatory properties of sweet cherry fruit have been reported also by other authors, who suggested that polyphenols, especially anthocyanins, may be responsible for this effect [51,52]. Additionally, sweet cherry, especially leaves, are a rich source of myricetin [16], which is known from strong anti-inflammatory activity. In our study, the effect of dietary treatment on HO-1 as well as IL-6 level was not observed. We suppose that polyphenols contained in fruit and leaves of sweet cherry affect other inflammatory pathways. Furthermore, it was previously reported that Ppar-α affects not only lipid metabolism, but also proinflammatory pathways, through inhibiting the NF-κB [53]. In addition, TNF-α is known both as proinflammatory marker and factor, which causes the lipid changes by activating the acetyl-CoA carboxylase and inhibiting the LPL activity. In the groups of rats receiving HFr diet enriched with fruit and leaves, reduced CRP level, as well as, simultaneously, increased expression of *Ppar-α*, decreased lipid content in the liver, and lower concentration of TG, in comparison to group fed with NC diet, were observed. It suggests that polyphenolic compounds from fruit and leaves of sweet cherry, by affecting the expression of selected genes, regulate the various pathways, exerting a pleiotropic effect.

Despite the fact that the fruits are rich in fructose, they contain a large amount of bioactive compounds which can beneficially affect organism and counteract adverse effects of fructose added to food products. As shown in this study, one of these fruits is sweet cherry. On the other hand, it is worth to mention that the consumption of fruits, especially dried and lyophilized, should be rational and should be considered as a part of balanced diet.

## 5. Conclusions

In conclusion, sweet cherry fruit and leaves reduced the adverse changes arising from the consumption of high fructose diet. Intake of these parts of sweet cherry resulted in the improvement of liver function, as well as beneficially affected lipid metabolism, among others, by regulating the expression of genes associated with fatty acid synthesis and β-oxidation. Additionally, they exhibited the antioxidant and anti-inflammatory properties. Our results indicate that not only commonly consumed fruits, but also leaves can be potentially used as functional foods. These findings may be helpful in prevention and treatment of obesity-related metabolic diseases, especially cardiovascular diseases.

## Figures and Tables

**Table 1:** The composition of experimental diets.

Ingredient	Group					
	NC	HFr	HFr + F5%	HFr + F10%	HFr + L1%	HFr + L3%
	g/kg					
Corn Starch	532.486	0	0	0	0	0
Sucrose	100	0	0	0	0	0
Fructose	0	632.486	586.586	540.786	627.286	616.986
Casein	200	200	200	200	200	200
Soybean Oil	70	70	70	70	70	70
Fiber	50	50	45.9	41.7	45.2	35.5
Mineral Mix	35	35	35	35	35	35
Vitamin Mix	10	10	10	10	10	10
Leaves of sweet cherry	0	0	0	0	10	30
Fruits of sweet cherry	0	0	50	100	0	0
Choline	2.5	2.5	2.5	2.5	2.5	2.5
Tert-Butylhydroquinone	0.014	0.014	0.014	0.014	0.014	0.014

NC—negative control; HFr—high fructose; HFr + F5%—HFr diet with addition of 5% of fruits; HFr + F10%—HFr diet with addition of 10% of fruits; HFr + L1%—HFr diet with addition of 1% of leaves; HFr + L3%—HFr diet with addition of 3% of leaves.

**Table 2:** Body and organs weights, crude lipid content in the organs and selected serum biochemical parameters of experimental rats.

Parameter	Group					
	NC	HFr	HFr + F5%	HFr + F10%	HFr + L1%	HFr + L3%
Weight (g)						
Body weight gain	485.00 ± 47.10 a	465.50 ± 65.63 a	422.43 ± 38.26 a	465.25 ± 61.08 a	426.29 ± 66.31 a	479.00 ± 47.82 a
Liver	17.49 ± 2.20 a	24.39 ± 3.27 b	20.63 ± 3.64 ab	20.11 ± 2.12 a	19.62 ± 4.02 a	20.97 ± 4.02 ab
Heart	1.53 ± 0.13 ab	1.53 ± 0.17 ab	1.47 ± 0.11 a	1.64 ± 0.10 b	1.57 ± 0.17 ab	1.60 ± 0.10 ab
Kidneys *	3.11 ± 0.34 a	3.61 ± 0.40 bc	3.37 ± 0.27 ab	3.52 ± 0.38 bc	3.47 ± 0.28 ab	3.88 ± 0.35 c
Crude lipid content (g/100 g)						
Liver	15.27 ± 5.10 ab	19.50 ± 2.89 b	17.08 ± 4.41 ab	13.17 ± 3.04 a	12.45 ± 2.91 a	12.37 ± 5.28 a
Heart	8.18 ± 1.09 a	7.65 ± 0.64 a	8.75 ± 1.43 a	8.20 ± 1.07 a	8.01 ± 0.86 a	7.26 ± 0.93 a
Kidneys	12.65 ± 2.07 a	11.17 ± 0.81 a	12.41 ± 0.31 a	11.45 ± 0.92 a	11.15 ± 2.40 a	11.10 ± 1.60 a
Serum parameters						
Glucose (mg/dL) **	107 ± 16 a	101 ± 9 a	105 ± 6 a	99 ± 8 a	97 ± 14 a	98 ± 13 a
ALT (U/L)	15.41 ± 3.05 a	27.82 ± 6.15 d	21.87 ± 4.26 c	25.78 ± 5.11 d	17.44 ± 2.42 ab	19.86 ± 3.14 bc
AST (U/L)	30.54 ± 4.78 bc	33.34 ± 3.17 c	25.40 ± 3.41 a	31.65 ± 5.10 c	27.49 ± 2.62 ab	26.02 ± 5.52 a
TC (mmol/L)	3.15 ± 0.27 a	3.25 ± 0.34 a	3.21 ± 0.31 a	3.02 ± 0.22 a	3.18 ± 0.26 a	3.11 ± 0.39 a
HDL (mmol/L)	1.47 ± 0.21 ab	1.41 ± 0.15 a	1.60 ± 0.25 bc	1.48 ± 0.22 ab	1.66 ± 0.24 c	1.74 ± 0.22 c
LDL+VLDL (mmol/L)	0.69 ± 0.13 b	0.66 ± 0.20 b	0.58 ± 0.15 ab	0.53 ± 0.13 a	0.68 ± 0.18 b	0.56 ± 0.13 ab
TG (mmol/L)	2.32 ± 0.49 abc	2.69 ± 0.59 c	2.40 ± 0.52 bc	2.27 ± 0.47 ab	1.97 ± 0.43 a	2.34 ± 0.43 abc

NC—negative control; HFr—high fructose; HFr+F5%—HFr diet with addition of 5% of fruits; HFr + F10%—HFr diet with addition of 10% of fruits; HFr + L1%—HFr diet with addition of 1% of leaves; HFr + L3%—HFr diet with addition of 3% of leaves. * weight of both kidneys. ** in whole blood. Data are expressed as mean ± SD (*n* = 8). Values in the same rows with different letters (a–d) are statistically different (*p* < 0.05).

**Table 3:** Biochemical parameters related to oxidative stress and inflammation of experimental rats.

Parameter	Group					
	NC	HFr	HFr+F5%	HFr+F10%	HFr+L1%	HFr+L3%
Antioxidant status						
TAS (mmol Trolox)	0.83 ± 0.08 b	0.64 ± 0.07 a	0.66 ± 0.12 a	0.73 ± 0.05 ab	0.76 ± 0.09 ab	0.70 ± 0.10 a
SOD (U/mL) *	200.34 ± 18.45 bc	201.52 ± 19.36 bc	205.44 ± 15.52 c	201.16 ± 16.38 bc	188.93 ± 17.16 b	168.43 ± 16.90 a
GPx (U/mL) **	114.88 ± 14.37 a	111.57 ± 9.24 a	127.10 ± 13.17 b	147.95 ± 11.63 c	145.18 ± 14.44 c	138.95 ± 15.04 c
GR (U/L)	34.59 ± 5.33 a	36.64 ± 6.01 ab	42.86 ± 10.08 bc	43.34 ± 7.94 c	42.32 ± 9.41 bc	38.65 ± 5.64 abc
CAT (U/L)	15.87 ± 3.50 c	19.59 ± 2.20 d	15.64 ± 2.80 c	13.02 ± 2.43 ab	13.92 ± 2.74 bc	11.35 ± 1.86 a
HO-1 (µmol/L)	0.60 ± 0.29 a	0.55 ± 0.13 a	0.43 ± 0.13 a	0.57 ± 0.20 a	0.60 ± 0.10 a	0.56 ± 0.18 a
TBARS (nmol/mL)	4.22 ± 0.49 ab	4.44 ± 0.78 b	3.21 ± 0.82 a	3.37 ± 0.72 ab	3.44 ± 0.31 ab	4.24 ± 0.31 ab
Inflammation						
CRP (ng/mL)	78.82 ± 14.26 a	115.79 ± 14.83 b	78.72 ± 14.80 a	80.37 ± 15.23 a	70.64 ± 10.21 a	80.77 ± 12.04 a
IL-6 (ng/mL)	14.83 ± 1.33 a	14.80 ± 0.47 a	14.81 ± 0.40 a	14.51 ± 0.24 a	14.94 ± 1.03 a	14.94 ± 0.48 a
TNF-α (ng/mL)	11.25 ± 0.57 a	11.35 ± 0.33 a	11.45 ± 0.73 a	11.00 ± 0.30 a	11.42 ± 1.00 a	11.03 ± 0.53 a
IL-10 (ng/mL)	8.63 ± 0.98 a	9.43 ± 1.00 a	9.88 ± 1.53 ab	12.28 ± 2.49 c	9.92 ± 1.26 ab	11.91 ± 0.88 bc

NC—negative control; HFr—high fructose; HFr + F5%—HFr diet with addition of 5% of fruits; HFr + F10%—HFr diet with addition of 10% of fruits; HFr + L1%—HFr diet with addition of 1% of leaves; HFr + L3%—HFr diet with addition of 3% of leaves. * in erythrocyte lysate; ** in whole blood. Data are expressed as mean ± SD (*n* = 8). Values in the same rows with different letters (a–d) are statistically different (*p* < 0.05).

## Supplementary Materials

The following are available online at https://www.mdpi.com/2072-6643/11/11/2638/s1. Table S1: The fatty acid profile in the liver of experimental rats, Table S2: The fatty acid profile in the kidneys of experimental rats, Table S3: The fatty acid profile in the heart of experimental rats, Table S4: The fatty acid profile in the adipose tissue of experimental rats.
