# Supplementary material for: High-Fructose Diet-Induced Metabolic Disorders Were Counteracted by the Intake of Fruit and Leaves of Sweet Cherry in Wistar Rats
Source: Nutrients. 2019 Nov 3;11(11):2638. doi: 10.3390/nu11112638 (PMC6893591; doi:10.3390/nu11112638)
Supplement: Supplementary file 1 [file nutrients-11-02638-s001.pdf]

Table 1S. The fatty acid profile in the liver of experimental rats.

Fatty acid	Group					
	NC	HFr	HFr+F5%	HFr+F10%	HFr+L1%	HFr+L3%
	%					
C14:0	1.15 ± 0.25 a	1.08 ± 0.01 a	0.80 ± 0.19 a	0.77 ± 0.13 a	0.86 ± 0.21 a	0.92 ± 0.18 a
C14:1	0.12 ± 0.01 c	0.11 ± 0.01 c	0.08 ± 0.01 b	0.05 ± 0.01 a	0.09 ± 0.01 b	0.09 ± 0.01 b
C15:0	0.10 ± 0.01 ab	0.09 ± 0.04 a	0.09 ± 0.01 a	0.16 ± 0.02 b	0.06 ± 0.02 a	0.08 ± 0.03 a
C16:0	32.82 ± 2.43 a	31.86 ± 3.94 a	30.31 ± 1.61 a	29.47 ± 1.67 a	31.85 ± 0.18 a	31.07 ± 3.42 a
C16:1	10.76 ± 0.98 a	6.19 ± 0.30 a	7.75 ± 2.88 a	7.66 ± 0.40 a	9.77 ± 2.47 a	8.81 ± 2.38 a
C17:0	0.19 ± 0.03 a	0.20 ± 0.02 a	0.23 ± 0.09 a	0.23 ± 0.08 a	0.17 ± 0.06 a	0.30 ± 0.04 a
C17:1	0.18 ± 0.02 a	0.12 ± 0.04 a	0.16 ± 0.01 a	0.14 ± 0.04 a	0.19 ± 0.06 a	0.17 ± 0.01 a
C18:0	6.09 ± 0.18 a	8.36 ± 2.81 a	5.75 ± 2.14 a	7.67 ± 0.08 a	6.48 ± 1.25 a	7.11 ± 1.56 a
C18:1 (n-9)	28.39 ± 2.4 a	33.01 ± 5.93 a	35.91 ± 2.37 a	35.38 ± 1.29 a	35.44 ± 1.00 a	36.19 ± 4.47 a
C18:2 (n-6)	14.01 ± 5.00 a	10.30 ± 4.17 a	13.02 ± 4.31 a	11.43 ± 0.8 a	9.76 ± 1.78 a	9.39 ± 3.29 a
C18:3 (n-6)	1.29 ± 0.23 c	0.74 ± 0.12 ab	0.69 ± 0.28 ab	0.97 ± 0.12 bc	0.37 ± 0.08 a	0.41 ± 0.08 a
C20:3 (n-6)	0.13 ± 0.04 a	0.21 ± 0.03 b	0.18 ± 0.01 ab	0.24 ± 0.02 b	0.22 ± 0.04 b	0.34 ± 0.04 c
C20:4	3.62 ± 1.53 a	6.16 ± 2.20 a	3.30 ± 0.46 a	3.82 ± 0.09 a	3.31 ± 0.28 a	4.34 ± 1.60 a
C24:0	0.36 ± 0.06 ab	1.31 ± 0.18 c	0.35 ± 0.03 a	0.59 ± 0.08 b	0.53 ± 0.03 ab	0.48 ± 0.04 ab
unidentified	0.82 ± 0.46	0.29 ± 0.64	1.42 ± 0.88	1.47 ± 4.16	0.94 ± 0.50	0.35 ± 3.78
SFA	40.71 ± 2.47 ab	42.89 ± 0.89 b	37.52 ± 0.28 a	38.88 ± 1.85 ab	39.93 ± 0.86 ab	39.94 ± 1.92 ab
MUFA	39.44 ± 3.42 a	39.42 ± 6.26 a	43.89 ± 5.28 a	43.22 ± 1.75 a	45.49 ± 3.54 a	45.25 ± 6.87 a
PUFA	19.04 ± 6.34 a	17.40 ± 6.52 a	17.17 ± 4.12 a	16.44 ± 0.57 a	13.66 ± 2.18 a	14.47 ± 5.01 a

SFA—saturated fatty acids; MUFA—monounsaturated fatty acids; PUFA—polyunsaturated fatty acids
Data are expressed as mean ± SD (n = 8).
Values in the same rows with different letters (a–c) are statistically different ($p < 0.05$).

Table 2S. The fatty acid profile in the kidneys of experimental rats.

Fatty acid	Group					
	NC	HFr	HFr+F5%	HFr+F10%	HFr+L1%	HFr+L3%
	%					
C12:1	0.24 ± 0.02 a	0.25 ± 0.03 a	0.34 ± 0.06 a	0.32 ± 0.09 a	0.34 ± 0.15 a	0.36 ± 0.13 a
C14:0	0.97 ± 0.09 a	0.95 ± 0.01 a	0.94 ± 0.05 a	0.78 ± 0.04 a	0.88 ± 0.09 a	0.87 ± 0.19 a
C14:1	0.13 ± 0.01 ab	0.21 ± 0.01 c	0.14 ± 0.04 ab	0.12 ± 0.01 a	0.19 ± 0.01 b	0.12 ± 0.02 a
C14:2	0.14 ± 0.01 ab	0.11 ± 0.02 a	0.11 ± 0.02 a	0.15 ± 0.01 b	0.15 ± 0.01 b	0.12 ± 0.01 ab
C15:0	0.17 ± 0.01 d	0.09 ± 0.01 bc	0.06 ± 0.01 ab	0.08 ± 0.02 bc	0.07 ± 0.00 abc	0.04 ± 0.00 a
C16:0	26.1 ± 0.84 b	23.06 ± 1.2 a	24.33 ± 1.70 ab	24.51 ± 0.28 ab	22.77 ± 0.24 a	23.78 ± 0.30 ab
C16:1	5.92 ± 0.74 a	4.77 ± 0.50 a	5.24 ± 0.50 a	4.71 ± 0.22 a	4.19 ± 1.17 a	4.43 ± 0.62 a
C17:0	0.29 ± 0.05 a	0.26 ± 0.05 a	0.28 ± 0.09 a	0.23 ± 0.02 a	0.27 ± 0.02 a	0.25 ± 0.01 a
C17:1	0.17 ± 0.01 bc	0.19 ± 0.01 c	0.16 ± 0.04 bc	0.18 ± 0.01 c	0.11 ± 0.01 a	0.13 ± 0.01 ab
C18:0	12.43 ± 0.83 a	13.24 ± 0.23 a	12.17 ± 0.76 a	13.20 ± 1.89 a	13.00 ± 1.67 a	13.3 ± 0.45 a
C18:1 (n-9)	29.3 ± 1.10 b	25.84 ± 0.54 ab	27.35 ± 1.31 ab	27.59 ± 2.63 ab	26.51 ± 2.30 ab	24.71 ± 1.64 a
C18:2 (n-6)	13.59 ± 1.22 a	16.32 ± 0.03 bc	15.54 ± 1.15 abc	15.39 ± 0.99 ab	17.54 ± 0.04 c	15.62 ± 0.18 abc
C18:3 (n-6)	0.74 ± 0.16 a	0.98 ± 0.01 abc	1.16 ± 0.09 c	0.81 ± 0.19 ab	1.07 ± 0.10 bc	0.87 ± 0.11 abc
C20:2 (n-6)		0.17 ± 0.01 a	0.17 ± 0.01 a	0.18 ± 0.01 a	0.16 ± 0.04 a	0.16 ± 0.01 a
C20:3 (n-3)	1.17 ± 0.10 b	0.20 ± 0.04 a	0.21 ± 0.01 a	0.23 ± 0.01 a	0.31 ± 0.05 a	0.30 ± 0.04 a
C20:4 (n-6)	7.55 ± 0.69 a	12.39 ± 0.57 b	11.18 ± 0.84 ab	10.82 ± 1.74 ab	12.02 ± 1.72 b	12.80 ± 3.07 b
C24:0	0.27 ± 0.02					
C20:5 (n-3)	0.09 ± 0.00 a	0.16 ± 0.01 c	0.08 ± 0.01 a	0.13 ± 0.02 b	0.17 ± 0.00 c	0.17 ± 0.00 c
C22:6	0.22 ± 0.01 b	0.20 ± 0.06 ab	0.22 ± 0.04 b	0.21 ± 0.01 b	0.12 ± 0.01 a	0.12 ± 0.01 a
unidentified	0.55 ± 0.28	0.65 ± 0.02	0.40 ± 0.29	0.41 ± 1.36	0.17 ± 0.30	1.88 ± 0.57
SFA	40.21 ± 0.08 a	37.59 ± 1.51 a	37.76 ± 2.40 a	38.78 ± 1.57 a	36.98 ± 1.32 a	38.24 ± 0.02 a
MUFA	35.75 ± 1.80 a	31.26 ± 0.98 a	33.21 ± 1.95 a	32.91 ± 2.76 a	31.32 ± 3.32 a	29.74 ± 2.35 a
PUFA	23.50 ± 2.16 a	30.51 ± 0.50 b	28.64 ± 0.16 b	27.90 ± 2.55 ab	31.53 ± 1.70 b	30.15 ± 2.94 b

SFA—saturated fatty acids; MUFA—monounsaturated fatty acids; PUFA—polyunsaturated fatty acids

Data are expressed as mean ± SD (n = 8).

Values in the same rows with different letters (a–d) are statistically different ($p < 0.05$).

Table 3S. The fatty acid profile in the heart of experimental rats.

Fatty acid	Group					
	NC	HFr	HFr+F5%	HFr+F10%	HFr+L1%	HFr+L3%
	%					
C12:1	0.35 ± 0.03 a	0.59 ± 0.03 b	0.36 ± 0.00 a	0.66 ± 0.11 b	0.56 ± 0.08 b	0.41 ± 0.01 a
C14:0	0.95 ± 0.04 ab	0.81 ± 0.25 ab	1.08 ± 0.07 b	0.81 ± 0.06 ab	0.66 ± 0.06 a	0.99 ± 0.13 ab
C14:1	0.14 ± 0.00 a	0.14 ± 0.04 a	0.18 ± 0.06 a	0.15 ± 0.04 a	0.19 ± 0.00 a	0.14 ± 0.02 a
C14:2	0.24 ± 0.01 c	0.19 ± 0.04 bc	0.13 ± 0.04 ab	0.17 ± 0.02 b	0.13 ± 0.02 ab	0.10 ±0.01 a
C15:0	0.14 ± 0.03 a	0.26 ± 0.03 b	0.06 ± 0.01 a	0.07 ± 0.01 a	0.31 ± 0.11 b	0.29 ± 0.01 b
C16:0	21.64 ± 1.63 a	19.35 ± 1.66 a	21.5 ± 1.42 a	20.04 ± 1.07 a	19.9 ± 0.58 a	20.72 ± 0.81 a
C16:1	4.11 ± 0.10 ab	4.24 ± 0.00 b	4.43 ± 0.91 b	3.12 ± 0.91 ab	2.52 ± 0.75 a	3.80 ± 0.56 ab
C17:0	0.23 ± 0.02 a	0.22 ± 0.01 a	0.19 ± 0.01 a	0.21 ± 0.03 a	0.19 ± 0.04 a	0.19 ± 0.02 a
C17:1	0.16 ± 0.03 ab	0.18 ± 0.01 ab	0.14 ± 0.01 a	0.18 ± 0.01 abc	0.23 ± 0.01 c	0.20 ± 0.03 bc
C18:0	18.13 ± 2.33 a	16.21 ± 0.45 a	14.1 ± 1.97 a	15.64 ± 0.38 a	17.01 ± 2.70 a	16.67 ± 1.48 a
C18:1 (n-9)	24.38 ± 1.25 a	25.23 ± 2.57 a	27.74 ± 1.98 a	26.55 ± 0.33 a	26.54 ± 3.94 a	26.77 ± 1.59 a
C18:2 (n-6)	18.93 ± 0.76 a	19.75 ± 2.26 a	19.7 ± 1.08 a	19.56 ± 0.62 a	20.50 ± 0.73 a	19.79 ± 1.37 a
C18:3 (n-6)	1.08 ± 0.08 ab	0.91 ± 0.25 a	1.29 ± 0.01 b	1.84 ± 0.08 c	0.83 ± 0.13 a	0.93 ± 0.15 a
C20:2 (n-6)	0.27 ± 0.06 c	0.15 ± 0.00 b	0.16 ± 0.00 b	0.93 ± 0.00 d	0.07 ± 0.00 a	0.20 ± 0.01 b
C20:4 (n-6)	7.86 ± 2.45 a	9.48 ± 1.32 a	6.91 ± 1.03 a	7.72 ± 1.36 a	8.79 ± 1.56 a	7.67 ± 0.33 a
C20:5	0.53 ± 0.00 c	0.20 ± 0.04 ab	0.27 ± 0.06 ab	0.33 ± 0.11 b	0.21 ± 0.03 ab	0.18 ± 0.00 a
C22:6	0.42 ± 0.13 a	2.00 ± 0.25 c	1.23 ± 0.25 b	1.23 ± 0.43 b	1.36 ± 0.27 b	0.99 ± 0.01 ab
unidentified	0.46 ± 2.14	0.12 ± 0.14	0.56 ± 0.15	0.82 ± 0.92	0.04 ± 0.01	0.01 ± 0.01
SFA	41.09 ± 4.05 a	36.84 ± 1.43 a	36.92 ± 0.47 a	36.76 ± 1.47 a	38.06 ± 2.21 a	38.84 ± 0.55 a
MUFA	29.14 ± 1.29 a	30.37 ± 2.51 a	32.85 ± 2.95 a	30.66 ± 0.67 a	30.03 ± 4.62 a	31.31 ± 2.11 a
PUFA	29.32 ± 3.20 a	32.68 ± 4.08 a	29.68 ± 2.33 a	31.77 ± 1.22 a	31.87 ± 2.42 a	29.84 ± 1.57 a

SFA—saturated fatty acids; MUFA—monounsaturated fatty acids; PUFA—polyunsaturated fatty acids

Data are expressed as mean ± SD (n = 8).

Values in the same rows with different letters (a–d) are statistically different ($p < 0.05$).

Table 4S. The fatty acid profile in the adipose tissue of experimental rats.

Fatty acid	Group					
	NC	HFr	HFr+F5%	HFr+F10%	HFr+L1%	HFr+L3%
			%			
C14:0	1.20 ± 0.01 a	1.14 ± 0.11 a	1.11 ± 0.09 a	1.02 ± 0.05 a	1.02 ± 0.15 a	1.09 ± 0.03 a
C15:0	0.06 ± 0.01 a	0.07 ± 0.01 a	0.08 ± 0.02 a	0.08 ± 0.03 a	0.09 ± 0.01 a	0.10 ± 0.01 a
C16:0	27.38 ± 0.13 a	24.35 ± 1.57 a	23.67 ± 2.14 a	23.32 ± 0.92 a	23.95 ± 0.87 a	24.24 ± 2.79 a
C16:1	7.53 ± 0.42 a	8.07 ± 0.49 a	6.90 ± 0.59 a	6.16 ± 1.20 a	6.81 ± 1.59 a	7.49 ± 0.07 a
C17:0	0.11 ± 0.00 a	0.15 ± 0.04 ab	0.19 ± 0.01 ab	0.17 ± 0.04 ab	0.21 ± 0.01 b	0.20 ± 0.06 ab
C17:1	0.21 ± 0.06 a	0.17 ± 0.01 a	0.17 ± 0.01 a	0.16 ± 0.01 a	0.22 ± 0.06 a	0.15 ± 0.01 a
C18:0	3.55 ± 0.34 a	3.03 ± 0.94 a	2.67 ± 0.20 a	2.67 ± 0.40 a	2.53 ± 0.68 a	2.65 ± 0.72 a
C18:1 (n-9)	33.31 ± 0.85 a	38.61 ± 0.19 b	36.45 ± 1.34 b	37.14 ± 1.49 b	37.99 ± 1.99 b	36.57 ± 0.49 b
C18:2 (n-6)	24.59 ± 0.80 a	22.44 ± 1.65 a	26.29 ± 3.51 a	26.81 ± 1.31 a	25.2 ± 2.01 a	24.96 ± 3.53 a
C18:3 (n-6)	2.01 ± 0.18 a	1.88 ± 0.06 a	2.45 ± 0.46 a	2.45 ± 0.23 a	2.01 ± 0.02 a	2.48 ± 0.45 a
unidentified	0.08 ± 0.01	0.12 ± 0.08	0.05 ± 0.01	0.05 ± 0.02	0.00 ± 0.00	0.09 ± 0.01
SFA	32.29 ± 0.21 a	28.73 ± 2.35 a	27.70 ± 2.02 a	27.26 ± 1.28 a	27.79 ± 1.40 a	28.28 ± 3.40 a
MUFA	41.04 ± 1.20 a	46.84 ± 0.68 b	43.52 ± 1.94 ab	43.45 ± 0.28 ab	45.01 ± 3.52 ab	44.20 ± 0.57 ab
PUFA	26.60 ± 0.98 a	24.32 ± 1.59 a	28.73 ± 3.97 a	29.25 ± 1.54 a	27.21 ± 2.03 a	27.43 ± 3.97 a

SFA—saturated fatty acids; MUFA—monounsaturated fatty acids; PUFA—polyunsaturated fatty acids

Data are expressed as mean ± SD (n = 8).

Values in the same rows with different letters (a–b) are statistically different ($p < 0.05$).
